# Cardiac Sarcoidosis as an Uncommon Etiology for Posterior Circulation Stroke Presenting with Alexia without Agraphia

**DOI:** 10.1155/2018/3418465

**Published:** 2018-12-17

**Authors:** Mohankumar Kurukumbi, Lauren Gardiner, Shevani Sahai, John W. Cochran

**Affiliations:** ^1^Department of Neurology, Inova Fairfax Hospital, Falls Church, VA, USA; ^2^VCU School of Medicine, Inova Campus, Falls Church, VA, USA

## Abstract

Sarcoidosis is a systemic disease with cardiac involvement occurring in 20-50% of cases. Cardiogenic stroke caused by cardiac sarcoidosis, especially PCA infarction, is a rare clinical presentation that necessitates timely diagnosis and may warrant treatment prophylaxis against CVA. In this case report, we describe a 54-year-old Caucasian male presenting with left PCA stroke in the setting of cardiac and pulmonary sarcoidosis, and hypertension. His presenting symptoms included right partial hemianopia, difficulty with naming, memory, and recall, and alexia without agraphia. Cardiogenic stroke is an uncommon manifestation of cardiac sarcoidosis, and given the disabling nature of these sequelae, the importance of early diagnosis and prevention with anticoagulation is crucial to prevent morbidity and mortality.

## 1. Introduction

Cardiac sarcoidosis is a rare manifestation of systemic sarcoidosis in which noncaseating granulomas infiltrate myocardial tissue resulting in ventricular wall motion abnormalities, life-threatening arrhythmias, and heart failure. Here, we present a rare case of left PCA infarction in the setting of cardiac sarcoidosis.

## 2. Case Presentation

Our patient is a 54-year-old Caucasian male with a history of cardiac and pulmonary sarcoidosis, hypertension, premature ventricular contractions (PVCs), and obesity who presented with acute onset right hemianopsia, memory recall difficulty, and alexia without agraphia. He was in his normal state of health and doing yard work when the symptoms began.

In the past year, incidental PVCs were found on 12-lead electrocardiogram (ECG) during a preoperative evaluation for dental work. Holter monitoring revealed a 12% PVC burden over 24 hours, indicating an indeterminate degree of ventricular dysfunction. Cardiac evaluation of the PVCs included transthoracic echocardiogram which revealed hypokinesis of the left inferior ventricular wall with an ejection fraction of 35%. Cardiac catheterization for investigation of structural blockages of coronary vessels yielded no significant CAD.

These findings were suspicious for an infiltrative process. This hypothesis was supported by cardiac MRI showing sarcoid infiltrates on T2-weighted images and by discovery of noncaseating granulomas on pulmonary node biopsy. He was diagnosed with cardiac sarcoidosis three months after initial presentation. During this time, he showed no clinical symptoms of systemic sarcoidosis or heart failure. An implantable cardioverter defibrillator was placed for primary prevention of arrhythmias secondary to cardiac sarcoid. He was doing well for one year until he presented with stroke symptoms.

Our patient endorsed decreased vision on the right and described the words on his lawn mower being visible but not readable. He also acknowledged trouble with recalling names and specific events.

Upon examination, our patient demonstrated normal speech and language. He was asked to write a simple sentence and performed the task without difficulty. When asked to read the sentence, he was unable to do so, representing alexia without agraphia. He showed right homonymous hemianopia. The remainder of the physical exam was normal.

**Figure 1:**
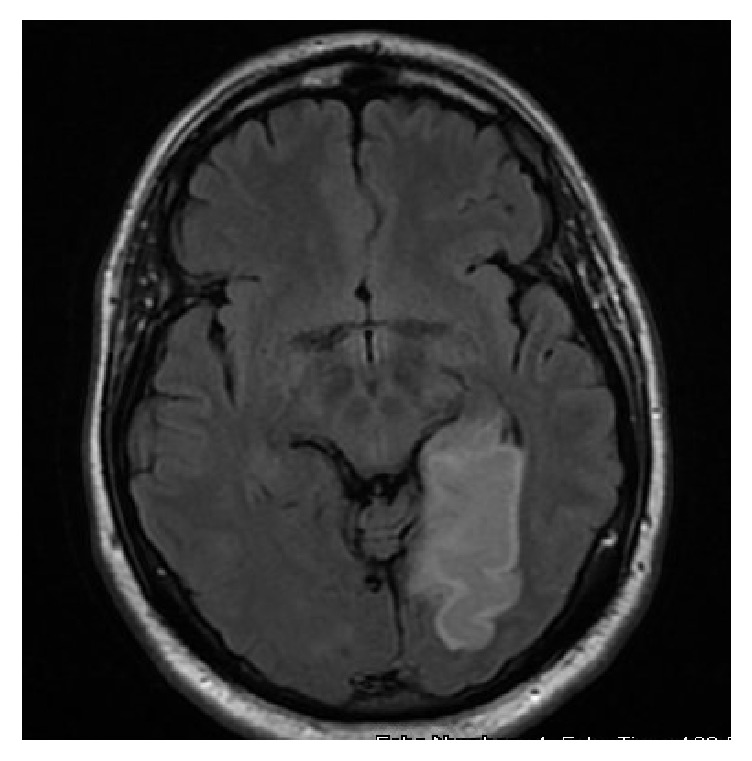
Brain MRI axial T2 FLAIR image showing left PCA infarction.

**Figure 2:**
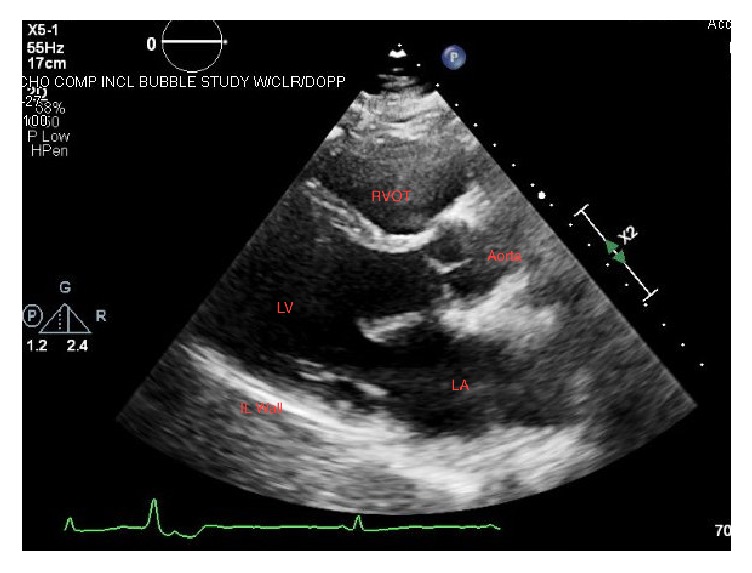
Transesophageal echocardiogram, parasternal long axis view. LA: left atrium. LV: left ventricle. RVOT: right ventricular outflow tract. IL wall: inferolateral left ventricular wall showing hypokinesis of ventricular myocardium.

At admission, initial computed tomography (CT) scan was negative. Subsequent brain magnetic resonance imaging (MRI) and magnetic resonance angiogram (MRA) confirmed a left posterior cerebral artery (PCA) infarction (Figure 1). MRA of the neck was unremarkable. The stroke was suspected to be cardioembolic in origin due the PVC burden and reduced ejection fraction of 35% promoting possible thrombus formation. Transesophageal echocardiogram revealed no thrombus or patent foramen ovale (PFO) and supported the previous finding of hypokinesis of left inferolateral ventricular wall (Figure 2). Hypercoagulable workup was nonrevealing. There was no family history of sarcoidosis or early age stroke.

By the time of discharge, our patient's visual symptoms returned to baseline and he was given high dose aspirin and atorvastatin for secondary stroke prophylaxis. Cellcept and prednisone were prescribed for management of sarcoidosis. A LINQ device was placed for continuous ECG monitoring. Lisinopril and metoprolol were maintained for pressure and rhythm control.

## 3. Discussion

Cardiac sarcoidosis is a rare manifestation of sarcoidosis that can occur in severe systemic disease or as an isolated characteristic of the condition. In the United States, up to 27% of patients with known systemic sarcoidosis have been reported to have cardiac sarcoidosis [1]. It is estimated that up to 25% of sarcoidosis related deaths are attributed to cardiac involvement. The hallmark of cardiac sarcoidosis is the presence of noncaseating granulomas involving myocardial tissue, with the most common location being within ventricular myocardium. Granulomas can range from small areas of inflammation to significant scarring. These lesions can progress to clinical symptoms such as atrioventricular block, arrhythmias, heart failure, stroke, and even sudden cardiac death [2]. The natural history of cardiac sarcoidosis is unpredictable and many patients experience a two-year delay in diagnosis [3].

The average age of diagnosis is 52 years and the most common presenting symptom is arrhythmia [l]. The first step to diagnosis involves taking a detailed history, clinical examination, and electrocardiography. Up to 85% of cases can be detected with this method alone in patients with known sarcoid disease [4]. However, advanced imaging with PET scans, MRI, and nuclear isotope perfusion scanning are generally performed to identify occult cardiac sarcoidosis.

Cardiac structural changes may lead to hypokinesis, stasis with clot formation, and stroke.

Occasionally, a cardiogenic embolism can travel through to the posterior circulation and affect distinct structures within the vascular territory. However, given the rarity of stroke secondary to cardiac sarcoidosis, little data currently exists regarding the most common localization of ischemia. There is no way to firmly attribute our patient's stroke to cardiac sarcoidosis, and the diagnosis remains embolic stroke of undetermined source (ESUS).

Alexia without agraphia is a syndrome involving impaired communication between the inferior portion of the splenium of the corpus callosum and the medial occipitotemporal gyrus of the dominant hemisphere [5]. This condition leaves patients with preserved visual recognition and writing skills, but impaired ability to read due to a loss of visual input to the language area of the left angular gyrus [6]. Alexia without agraphia is most commonly caused by PCA infarction.

The current management of cardiac sarcoidosis largely focuses on immunosuppression, arrhythmia prevention, and heart failure assessment. The primary goal of treatment is to decrease the risk sudden cardiac death and prevent granuloma progression to fibrosis [4]. Corticosteroids are the cornerstone of therapy and have been shown to improve survival [4]. Immunosuppressive agents, such as mycophenolate or methotrexate, are used to decrease the steroid dose and are considered second-line therapies. Our patient was treated with mycophenolate and prednisone.

There are no set guidelines suggesting anticoagulation as part of the standard treatment protocol for cardiac sarcoidosis [7]. Our current knowledge regarding anticoagulation therapy is shaped by the WARCEF study of 2012 and a concomitant subgroup analysis published in 2013. Conclusions from these studies suggest a limited role for anticoagulation in patients with reduced ejection fraction who are in normal sinus rhythm [8]. The 2013 substudy showed a benefit for warfarin over aspirin specifically in a subgroup of patients <60 years of age with combined outcomes of ischemic stroke, ICH, or death [9]. The 2012 WARCEF recommendations were followed for our patient who was not anticoagulated after the diagnosis of cardiac sarcoidosis with reduced ejection fraction of 35%.

## 4. Conclusions

This case is reported due to the rare constellation of clinical syndromes: cardiac sarcoidosis as a possible etiology of left PCA stroke resulting in right hemianopia and alexia without agraphia. Cardiogenic stroke is a poorly understood manifestation of cardiac sarcoidosis, and given the disabling nature of these sequelae, the importance of early diagnosis and prevention is crucial to prevent morbidity and mortality. The role of anticoagulation should be investigated in cardiac sarcoid patients.

## Abbreviations

ACA: Anterior cerebral artery
CAD: Coronary arterial disease
CT: Computed tomography
CVA: Cerebrovascular accident
ECG: Electrocardiogram
ESUS: Embolic stroke of undetermined significance
FLAIR: Fluid attenuated inversion recovery
MRA: Magnetic resonance angiogram
MRI: Magnetic resonance imaging
PCA: Posterior cerebral artery
PET: Positron emission tomography
PVC: Premature ventricular contractions.
